# Exploring community members’ perceptions of oral health in rural South Africa

**DOI:** 10.1186/s12903-025-07490-1

**Published:** 2025-12-07

**Authors:** Hlulani Alloy Nghayo, Khabiso Jemima Ramphoma, Ronel Maart

**Affiliations:** 1 Department of Community Dentistry, Faculty of Dentistry, University of the Western Cape, Cape Town, South Africa; 2 Department of Prosthodontics, Faculty of Dentistry, University of the Western Cape, Cape Town, South Africa; 3 Department of Sport, Rehabilitation and Dental Sciences, Faculty of Science, Tshwane University of Technology, Pretoria, South Africa

**Keywords:** Oral health, Oral health policy, Rural population, Primary health care, Health services accessibility, South africa

## Abstract

**Background:**

Oral health remains a global health challenge. Rural communities are disproportionately affected by oral diseases due to inequitable access to oral health services and the absence of initiatives that promote oral health and quality of life. This study aimed to explore community members’ perceptions of oral health in rural communities in Limpopo province, South Africa.

**Methods:**

A convenience sample of 50 participants was recruited to take part in five focus group discussions, each comprising 10 participants from five geographically distinct rural communities. Discussions were conducted using an interview guide, digitally recorded, transcribed verbatim, imported into ATLAS.ti, and thematically analyzed using an inductive approach.

**Results:**

Three main themes emerged: (1) barriers to oral healthcare access, (2) inadequate oral health advocacy, and (3) intrinsic determinants of oral health, each with several related sub-themes and categories. Together, these factors were identified as key contributors to limited awareness of oral health and its potential impact, thereby increasing the prevalence of oral diseases in rural communities.

**Conclusion:**

Despite global initiatives to improve oral health, rural communities remain disproportionately affected by various oral health challenges. Improving oral health in these settings requires integrating oral health into PHC policy reform, equitable workforce distribution, implementation of oral health programs, expansion of mobile services, and the involvement of community healthcare workers to enhance access.

**Supplementary Information:**

The online version contains supplementary material available at 10.1186/s12903-025-07490-1.

## Introduction

The World Health Organization (WHO) defines *oral health* as a state characterized by the absence of orofacial pain, oral or throat cancer, oral infections and lesions, periodontal diseases, dental caries, tooth loss, and other disorders that hinder an individual’s ability to chew, bite, express facial emotions, communicate verbally, and maintain psychosocial well-being [1]. Oral health is integral to physical, emotional, psychological, and socio-economic well-being across individual, interpersonal, community, and societal levels [2]. Despite its importance, it remains a major global public health concern, often receiving insufficient attention from policymakers and healthcare professionals [3]. The burden of oral diseases worldwide is considerable, with dental caries identified as the most prevalent condition, affecting approximately 3.5 billion people [4]. Current epidemiological data further show that dental caries in permanent teeth impact an estimated 2 billion individuals, while 520 million children experience dental caries in their primary teeth [5].

Although oral diseases are preventable through supportive environments, sufficient access to Primary Health Care (PHC) resources, adequate financial means, health-conscious behaviors, and greater oral health awareness [6, 7], rurality remains a key determinant of suboptimal oral health outcomes in rural communities [8–10]. Oral health inequalities and the underutilization of dental services have been extensively documented in both developing and developed countries [11–14]. However, the provision of these services in rural communities is hindered by complex challenges [12, 15], including poor infrastructure, inadequate public services, and inequitable allocation of health resources. Collectively, these barriers shape health perceptions, influence health-related behaviors, and restrict access to health care [12, 16–18].

Despite considerable progress in oral disease prevention, treatment, and overall oral health in recent years, a substantial disparity remains, mirroring inequalities observed in general health [19]. This ongoing discrepancy can be attributed to the consistent omission of rurality as a key factor in oral health policy development [10]. The failure to address this issue has perpetuated oral health disparities, including challenges related to distance and isolation that reinforce cultural barriers to healthcare access [20–22]; low income, which exacerbates costs such as transportation [15]; inequitable distribution of oral health professionals (OHPs) [23]; high treatment costs; declining private health insurance coverage [24, 25]; and limited access to oral health education [26, 27].

Rural populations in South Africa (SA) are highly diverse. The term “rural” refers not only to extensive commercial agricultural lands under freehold tenure, consistent with a traditional Western view, but also to dispersed communities located on communal lands, each with a distinct rural identity. These communal communities differ significantly from commercial farms in population density, land ownership, and socio-economic conditions [28]. Nevertheless, they face persistent barriers to accessing and using public healthcare services, with high-quality oral healthcare being particularly difficult to obtain [29]. For instance, Limpopo remains one of the most socio-economically disadvantaged provinces in SA, where an estimated 67.5% of the rural population lives in poverty [30].

This province lacks policies for implementing oral healthcare and strategies for prevention and promotion. Moreover, oral health advocacy is underutilized in health decision-making across the public health system, and oral health targets are often unrealistic due to insufficient legislation, support, and funding. The limited oral health services that do exist primarily benefit privileged and metropolitan areas [31], with dental extractions serving as the predominant – if not sole – service offered [32, 33]. The situation is further aggravated by shortages of dental equipment, materials, and supplies, alongside poor maintenance of mobile dental trucks, particularly those designated for rural community outreach. Adding to these challenges, a significant migration of community dentistry specialists from the province to urban cities has further weakened service delivery [34, 35].

To address healthcare shortages in rural communities, the WHO has proposed three key initiatives: educational and regulatory measures, financial incentives and management, and environmental and social support [36]. In addition, the WHO has also launched oral healthcare programs specifically targeting rural populations, integrating oral health education with preventative services, restorative treatments, and emergency dental interventions [37]. These efforts align with Several Sustainable Development Goals (SDGs), particularly good health and well-being, quality education, reduced inequality, and partnerships to achieve the goal. The SDGs are interconnected, recognizing that progress in one domain influences others, and that sustainable development requires balancing social, economic, and environmental priorities to fulfill the universal call to end poverty, protect the planet, and promote peace and prosperity by 2030 [38]. Collectively, these initiatives hold significant potential to advance global oral health objectives and contribute to the realization of universal health coverage [39, 40].

Global studies have consistently identified a range of strategies to mitigate disparities in oral health service accessibility. These include preventive and promotional public health initiatives, such as school-based interventions; enhancement of infrastructure and technological capacity through e-health solutions; provision of temporary care via mobile clinics; adoption of interdisciplinary models that integrate oral health into primary care; and academic initiatives, including rural-focused training programs and targeted recruitment efforts [8, 41–43]. Despite these measures, rural communities remain heavily burdened by oral diseases, owing to the absence of programs that integrate oral health professionals, alongside limited sustainability, and insufficient government support for resources to promote oral health [27].

The unique cultural and socio-economic context of rural SA requires a comprehensive examination of the factors shaping oral health attitudes and behavior. A qualitative research approach offers valuable potential to uncover the specific challenges rural populations face and to identify opportunities for advancing oral health promotion and policy development. Such an approach can provide critical insights to guide the design of contextually relevant policies and community engagement strategies, ensuring that oral health promotion and the integration of PHC more effectively address the needs of underserved populations. To the best of our knowledge, no qualitative study has investigated oral health in rural South African communities. In response, this study explored community members’ perceptions of oral health in rural communities in Limpopo province, SA.

## Methods

### Ethical considerations

Ethical approval for this study was granted by the Biomedical Research Ethics Committee of a higher education institution in the Western Cape province, Cape Town, SA (Reference: BM 23/6/16), and full compliance with the ethical standards set by the Declaration of Helsinki was ensured. Prior to each focus group discussion (FGD), information sheets outlining the study’s purpose were distributed and reviewed with all participants from the rural communities to ensure a clear understanding of its aims. Consent forms were then explained, distributed, and signed, with measures implemented to safeguard confidentiality. The principal author (HAN) collected the signed consent forms. Participants were reminded of their right to voluntary participation and informed that they could withdraw without penalty. To preserve anonymity, numerical identifiers were assigned to participants during the FGDs and used throughout the transcription and data analysis processes.

### Study design

This study employed a qualitative method using FGDs to conduct an exploratory descriptive analysis. The authors (HAN, KJR, and RM) selected FGDs as the most appropriate method for obtaining in-depth insights into participants’ perspectives on oral health. This method enabled the participants to engage in dialogue that revealed both explicit and implicit beliefs, as well as their perceptions and lived experiences [44, 45].

### Study setting

**Fig. 1:**
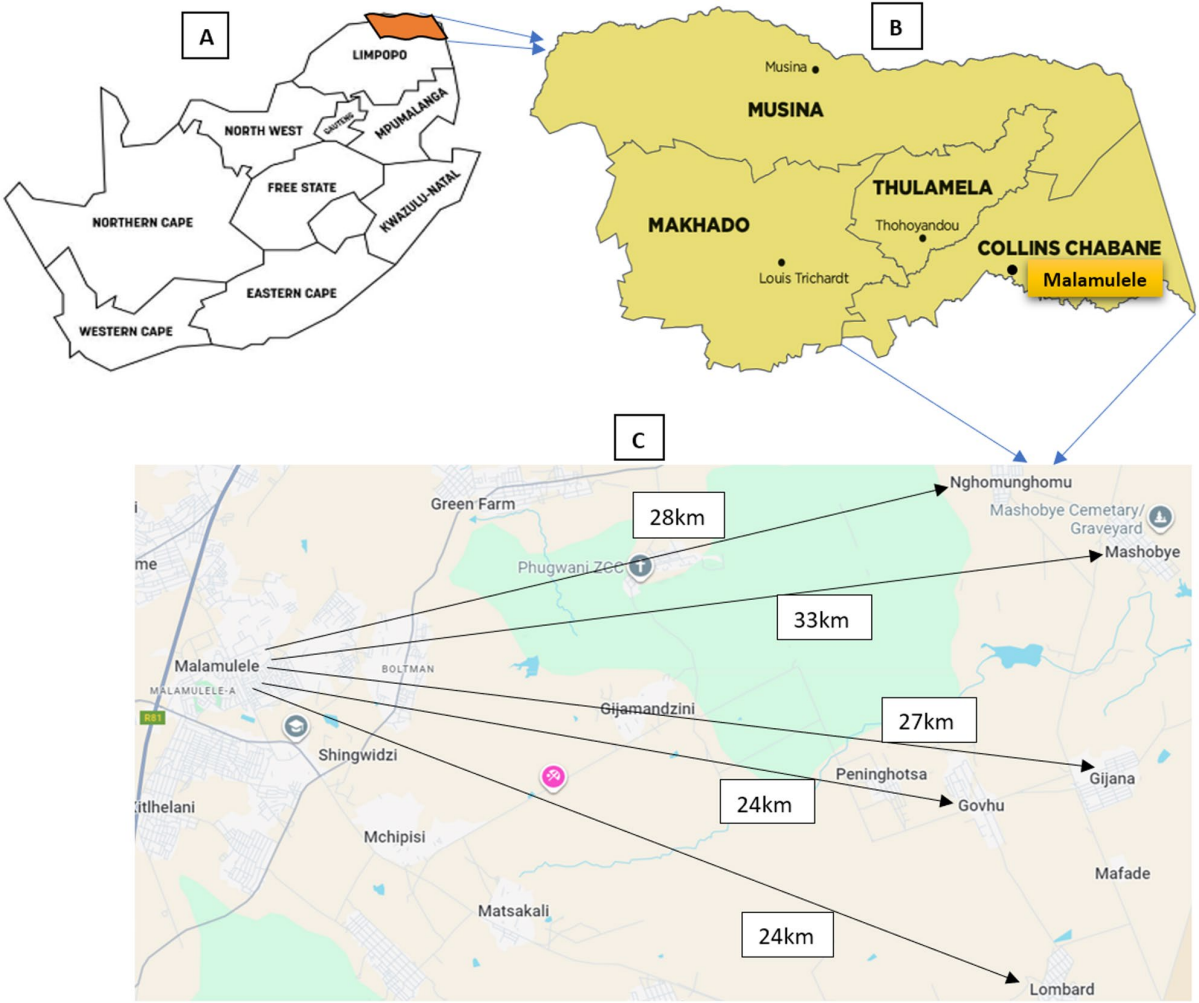
Study settings: (**A**) SA map, (**B**) Vhembe map [46], and (**C**) Google Maps (© Google, 2025)

The Vhembe District in Limpopo province, SA, comprises four local municipalities: Thulamela, Makhado, Musina, and Collins Chabane. Malamulele, a town within Collins Chabane and its administrative center, is predominantly inhabited by Tsonga people and is surrounded by approximately 120 rural villages. The rural communities surrounding Malamulele have a combined population of approximately 443,798. This study was conducted in five geographically distinct rural communities on the outskirts of Malamulele – Gijana (Magona), Govhu, Lombard, Mashobye, and Nghomunghomu – located, on average, 27 km from the town [46] **(**Fig. 1**)**.

The Malamulele area hosts a range of public healthcare facilities, including one hospital in town, three community health centers (CHCs), and 30 public PHC clinics dispersed across the villages. However, oral health services are provided exclusively at the hospital and a single CHC, delivered by a workforce of only four dentists and one oral hygienist [46]. This equates to one oral hygienist per 88,760 residents and one dentist per 110,950 residents – figures far below the WHO’s benchmarks and the national average of approximately one dentist per 8,000–9,000 people [1]. These deficits reveal profound inequities in workforce distribution that constrain access to oral healthcare in rural contexts [11, 23, 40]. Notably, the five study communities are each served by one public PHC clinic; however, this facility lacks the capacity to provide oral health services. Instead, they depend on intermittent visits from OHPs affiliated with the hospital located in town, further evidencing the structural barriers to equitable and continuous oral health service delivery in rural communities.

### Research team and reflexivity

The primary author (HAN), a male oral hygienist and dental educator with qualitative research training and clinical experience in rural oral health, conducted the FGDs. His prior residence in one of the selected communities enhanced cultural familiarity and rapport, fostering open dialogue, but also introduced the potential risk of shaping assumptions and interpretations. To mitigate this, he maintained reflexive awareness and triangulated his interpretations with co-authors who had no affiliation with the communities. Importantly, he had no prior personal relationship with participants, supporting neutrality. A community caregiver served as a research assistant, recording field notes, and documenting nonverbal cues to complement and balance the researcher’s perspective.

The second (KJR) and third (RM) female authors, both senior educators with extensive experience in community oral health, contributed to the analysis by offering complementary disciplinary and methodological perspectives, particularly in community engagement and mixed-methods research. Unlike the primary author, they were not affiliated with rural communities, which helped balance potential insider bias and strengthened the interpretation. All authors took part in iterative discussions throughout the analysis to maintain reflexivity, transparency, and credibility. The authors declare no conflicts of interest.

### Quality

To ensure transparency and methodological rigor, the study followed the Consolidated Criteria for Reporting Qualitative Research (COREQ) checklist [47]. The checklist was applied throughout the study design, data collection, and reporting to strengthen reflexivity, transparency, and rigor (S1 Text).

### Study population and recruitment procedure

A homogeneous convenience sampling approach was employed to recruit adult members from five rural communities, with participation facilitated through collaboration with community caregiver leaders. Caregivers play a pivotal role in establishing effective healthcare by providing essential support to individuals with functional limitations or diseases [48]. In these settings, caregiver leaders were instrumental in mobilizing residents; their involvement centered on awareness-raising and coordinating logistics, rather than determining eligibility. Eligibility criteria required participants to be permanent residents aged 18 years or older. Recruitment was designed to be inclusive, offering all eligible community members the opportunity to participate. Although caregiver leaders assisted with recruitment, the study is best characterized as using convenience sampling, as participation relied entirely on voluntary responses to open invitations. This approach was appropriate for capturing a broad range of community perspectives while reflecting the practical realities of rural recruitment.

Participants were recruited voluntarily through word of mouth, telephone invitations, posters, and flyers distributed within the communities. In total, 50 individuals participated (33 women and 17 men; aged 21–65 years, including 15 who had not completed secondary school). Five FGDs were conducted, with approximately 10 participants per community, ensuring inclusivity, manageability, and data saturation across sites [49]. The sample comprised unemployed youth, working-age adults, and community elders, providing a broad perspective on oral health practices and the challenges they face. The final composition of the groups was determined solely by the willingness of individuals who provided informed consent. FGDs were scheduled in collaboration with caregiver leaders to maximize accessibility and were conducted between 23 June and 19 July 2024. All sessions were completed by 1 August 2024.

### Data collection tool

The primary author developed a semi-structured interview guide (S2 Text) for data collection. The guide was informed by the established literature and adapted from previously validated frameworks [31, 50, 51]. The final version comprised 17 questions. These examined participants’ views on policies governing the implementation of oral health services, and their perceptions of how oral health experiences relate to the accessibility and delivery of such services. The primary author received training in qualitative interviewing techniques. A language expert translated the guide into Xitsonga (the national official language spoken in the communities). A pilot test with six members of a rural community not included in the main study assessed clarity, comprehension, and appropriateness. Subsequent revisions were made in response to feedback, primarily concerning question phrasing.

### Data collection process

The primary author conducted FGDs in five rural communities using a semi-structured interview guide with open-ended questions adapted from the literature. Each group was supported by a trained community caregiver leader whose contextual knowledge and cultural sensitivity enhanced the documentation of group dynamics, non-verbal cues, and related behaviors. Their involvement strengthened rapport, reduced the risk of misinterpretation, and contributed to the trustworthiness of the data through triangulation across sites.

At the beginning of each session, formal introductions were made, and participants were informed of the voluntary nature of their involvement and assured of confidentiality. Written informed consent was obtained before participation. The FGDs were held in local halls, audio-recorded with permission, and lasted approximately 45 min. Data saturation was achieved when no new concepts or perspectives emerged. All recordings and Xitsonga transcripts were subsequently translated into English in consultation with a language expert to ensure linguistic accuracy and cultural validity for analysis.

### Data analysis

The data were analyzed thematically using a combined approach that incorporated the general inductive method [52] and a three-stage process for qualitative data analysis [53]. The three-stage process, derived from Strauss and Corbin’s grounded theory [54], can be adapted to provide a systematic, theory-informed framework for coding and integrating data into core themes [55]. In this combined approach, the initial phase applied the general inductive method, involving a comprehensive review of FGD transcripts to identify and develop concepts and themes [52]. This step was carried out by all authors. The authors then used a modified version of the three-step coding and analysis process, consisting of open coding, axial coding, and selective coding [53, 56]. Verbatim transcripts were produced by an independent transcriber, who was not involved in data collection, ensuring a neutral and objective account of the discussions. These transcripts were verified against the audio recordings by the primary author, with non-verbal cues documented by research assistants incorporated to enrich the data. The transcripts were then imported into ATLAS.ti software (version 7.1.3) for analysis.

The primary and second authors independently coded the transcripts using descriptive labels informed by both the data and the literature. All authors then reviewed the coding frameworks, compared interpretations, and resolved discrepancies through consensus. To enhance credibility, an external coder conducted an independent coding process, which was cross-checked against the authors’ outputs to validate interpretations. Through this iterative and collaborative approach, the coding frameworks were consolidated into overarching thematic categories that reflected both shared and divergent perspectives across the FGDs. Each theme was supported with verbatim quotations to ensure transparency and grounding in participants’ voices.

### Trustworthiness

This study adhered to Lincoln and Guba’s four criteria of trustworthiness: credibility, transferability, dependability, and confirmability to ensure confidence in its findings [57]. *Credibility*, referring to confidence in the truth of the data and the plausibility of the findings, was strengthened through triangulation across five rural communities, the involvement of trained community caregivers in documenting group dynamics and non-verbal cues, and transcript verification by selected participants (member checking). Peer debriefing with co-researchers and consultation with an independent qualitative expert further bolstered the rigor of these interpretations [58, 59]. *Transferability*, concerned with the applicability of findings to other contexts, was supported by rich contextual descriptions of the study setting, participants, and health system context, enabling readers to judge the relevance of the findings to similar rural and low-resource settings [58, 59]. *Dependability*, relating to the stability and consistency of the data over time, was addressed through transparent, systematic documentation of the research process, including detailed accounts of sampling, data collection, and analysis procedures [58, 59]. *Confirmability*, which aims to ensure that the findings reflect the participants’ voices rather than researcher bias, was advanced through triangulation, inclusion of verbatim quotations, and reflexive practices, with interpretations cross-checked by all authors and validated through peer review [58, 59]. Collectively, these strategies ensured that the findings were both trustworthy and grounded [60] in the lived realities of rural communities.

## Results

A total of 50 community members (33 women, 17 men), aged 21 to 65 years, participated in five FGDs. The sample included unemployed youth, working-age adults, and elders, with 15 participants who had not completed secondary school. This heterogeneity provided a broad representation of community perspectives on oral health practices and challenges in rural settings.

The analysis of social dynamics across the FGDs indicated that dependence on a singular public primary healthcare clinic significantly influenced shared experiences while simultaneously generating divergent viewpoints. Consensus was often expressed through non-verbal cues such as nodding and verbal affirmations, whereas disagreements manifested through interruptions, side conversations, or tonal shifts, which signified the negotiation of meaning within the group. Emotional and embodied responses, including silence, sighs, raised voices, and leaning forward to emphasize a point, were crucial for interpreting the significance of specific concerns. These interactional cues were systematically coded alongside verbal accounts to ensure that the analysis encompassed both explicit statements and the underlying dynamics of collective meaning-making.

The participation of trained community caregiver leaders, who meticulously documented non-verbal behaviors and group interactions, further enhanced the credibility of the findings through triangulation and strengthened confirmability by mitigating reliance on a single researcher’s interpretation. Data from the five FGDs were subsequently synthesized for thematic analysis, with themes presented individually for analytic clarity, despite their frequent interweaving during discussions. Therefore, three overarching themes emerged from the data analysis process: barriers to oral healthcare access, inadequate oral health advocacy, and intrinsic determinants of oral health (Table 1).

**Table 1:** Main themes and sub-themes

Main themes	Sub-themes	Categories
1. Barriers to oral healthcare access	1.1 Absence of OHPs 1.2 Absence of mobile oral health services	
	1.3 PHC challenges	1.3.1 Lack of healthcare facilities 1.3.2 Barriers to oral health service delivery in PHC 1.3.3 Challenges with the referral system
	1.4 Geographic and infrastructural challenges 1.5 Financial barriers to oral healthcare	
2. Inadequate oral health advocacy	2.1 Lack of policy recognition for rural oral health 2.2 Lack of community-based oral health programs 2.3 Narrow scope of community health campaign	
3. Intrinsic determinants of oral health	3.1 Knowledge of oral health self-care	3.1.1 Awareness of the link between oral and general health 3.1.2 Awareness of oral health conditions 3.1.3 Daily routine of oral care 3.1.4 Routine dental check-ups for oral health maintenance
	3.2 Attitudes on oral health self-care	3.2.1 Prevention of oral health diseases 3.2.2 Physical functionality and well-being 3.2.3 Social and psychological implications
	3.3 Practices of oral health self-care	3.3.1 Promoting good oral hygiene habits 3.3.2 Need for immediate oral healthcare

### Theme 1: access to oral healthcare

#### Sub-theme 1.1: absence of OHPs

Across the FGDs, the acute shortage of OHPs in PHC clinics and hospitals emerged as a central barrier to accessing care. Participants noted that this workforce deficit restricted the immediate availability of services and deepened structural inequities by disproportionately disadvantaging underserved rural populations. The absence of OHPs at the primary care level was viewed not only as a practical service gap but also as a symbolic marker of neglect, undermining the principle of equity in healthcare provision. This lack of local services compels rural residents to bear additional travel and financial burdens, further entrenching systemic barriers to care. As one participant observed:

*“Unfortunately*,* even in our clinic [public PHC clinic]*,* dentists are not available to provide oral health services*,* and we have to visit the hospital” (C1P2).*

#### Sub-theme 1.2: absence of mobile oral health services

Participants consistently highlighted the lack of mobile oral health services in their communities, a gap that has persisted across generations and left many residents unaware of these services. This absence reinforces oral health neglect and contrasts sharply with the visibility of other health initiatives, particularly HIV awareness campaigns, which dominate community programs. These accounts highlight how the systemic prioritization of certain health conditions perpetuates the marginalization of oral health in rural communities.

*“There are no mobile dental services that come to us*,* but there are mobile health programs that mainly focus on educating people about HIV” (C4P1).*

*“I was born in this village more than 50 years ago*,* and I have never seen a mobile dental service before. I do not even know what it looks like” (C3P6).*

#### Sub-theme 1.3: PHC challenges

##### Category 1.3.1: lack of healthcare facilities

Participants consistently highlighted the shortage of local healthcare facilities, noting that a single public PHC clinic often served multiple villages. This structural deficiency generated widespread dissatisfaction, as the lack of OHPs effectively excluded community members from accessing even basic care. For many, the absence of nearby services constrained care-seeking behavior and imposed excessive travel burdens and delays, particularly for those required to walk long distances to the nearest clinic.

*“We do not have a clinic [public PHC clinic] in our community*,* which means to travel to the nearest village where there is a clinic [public PHC clinic]…” (C5P3).*

*“We only have one clinic [public PHC clinic] that provide other health services to four neighboring villages…” (C1P9).*

##### Category 1.3.2: barriers to oral health service delivery in PHC

A recurring concern raised by participants was the inadequate oral health knowledge among PHC nurses, perceived as a critical barrier to accessing essential care in rural communities. Limited training for these providers constrained their ability to deliver even basic preventive and restorative services, leading to a focus on temporary pain management instead of comprehensive oral health care. This gap undermines early detection and prevention and reinforces the cycle of delayed treatment and referral.

*“For as long as it is related to dental issues*,* the nursing staff always write a referral letter to hospitals*,* even for minor issues” (C5P10).*

*“The nurses at our clinic [public PHC clinic] are only interested in other health problems*,* and their dental knowledge is very limited that they only provide pain relief medication” (C1P6).*

Study participants consistently described their awareness of oral health services as limited to extractions, reflecting a curative, surgical model of care. Although many expressed a desire to preserve their natural teeth and practice good oral hygiene, their accounts revealed a striking lack of preventive guidance or health promotion during primary care encounters. This gap between patient expectations and available services illustrates a systemic failure to integrate prevention into routine oral healthcare, perpetuating inequities in rural communities and compromising oral health. Participants’ narratives highlight missed opportunities for health providers to support long-term oral health maintenance and empower communities with the knowledge needed for preventive practices. One participant explained,

*“Although i made sure that i always brushed my teeth to keep them healthy*,* but the time i visited the hospital*,* the only service provided by the dentist was pulling out of my tooth without explain how the problem can be prevented” (C2P7).*

Another participant added:

*“Unfortunately*,* there is shortage of chats about dental information at the facilities*,* and they do not even provide guidance on preventing dental problems*,* all they do is to take out teeth” (C4P5).*

##### Category 1.3.3: challenges with the referral system

A recurring concern across the FGDs was widespread dissatisfaction with the referral system. Participants described the need to make multiple visits to local PHC clinics just to obtain referral letters for treatment at distant hospitals as both burdensome and inefficient. This procedural bottleneck not only delayed timely access to oral healthcare but also highlighted the lack of clear and consistent referral guidelines within the system. Participants suggested that referrals were sometimes used as a means to manage patient load rather than to streamline care.

One participant remarked:

*“It is very frustrating to repeatedly travel to the nearest village merely to obtain a referral letter to the hospital*,* and they seem to make these referrals to reduce long queues in the clinics [public PHC clinic]” (C3P4).*

Another stated:

*“We must travel to the nearest village clinic [public PHC clinic] to obtain referral letters*,* allowing us to gain access to dental care at a hospital*,* this process is not always easy and lack consistency” (C5P8).*

#### Sub-theme 1.4: geographic and infrastructural challenges

Geographical isolation emerged as a major barrier to oral healthcare in rural communities. Participants explained that the remoteness of their settlements required extensive travel to the nearest dental facility, a challenge compounded by poorly maintained road networks and reliance on unpaved roads. These conditions increased travel time, heightened transportation costs – particularly for households without reliable vehicles – and placed disproportionate burdens on vulnerable groups, such as older adults, whose urgent oral health needs could not be addressed locally and often required long, costly referrals. This sentiment is captured in the following excerpts:

*“Accessing dental care is challenging*,* primarily because of the distance that we need to travel*,* which is worsened by poor road conditions. We must navigate approximately 8 km of gravel roads to reach the nearest local clinic [public primary health care clinic] because our community lacks a local clinic [public PHC clinic]” (C5P2).*

*“Accessing dental care is extremely challenging due to gravel roads (poor road conditions) and distance. This means that for emergency dental care*,* we have to arrange our own transportation to the nearest village clinic [public primary health care clinic] to be referred to the hospital. It becomes a problem when elderly people experience dental pain*,* as immediate treatment is not available*,* as it is financially burdensome” (C3P5).*

#### Sub-theme 1.5: financial barriers to oral healthcare

Participants highlighted financial hardship as a significant barrier to oral healthcare access in rural communities, emphasizing the theme of entrenched socio-economic inequities within the healthcare system. They consistently described how the combined burden of high treatment costs and additional travel expenses to distant facilities limited their ability to seek timely and essential care. These economic barriers not only discourage service utilization but also exacerbate oral health disparities between wealthier and disadvantaged groups. As one participant observed,

*“Accessing dental care is challenging primarily because of financial constraints. Individuals who can afford private care often choose to visit private doctors because they provide a much faster alternative to dental-care services” (C4P1).*

Another added:

*“For us to get dental care*,* the amount of money spent is too much because we have to visit the local clinic [public PHC clinic] first*,* which is money*,* then go to the hospital*,* which is money as well. Getting a treatment at a hospital involves money. So*,* for one to just say*,* I have received dental care*,* means they have spent a lot of money that we do not have” (C5P9).*

### Theme 2: inadequate oral health advocacy

#### Theme 2.1: lack of policy recognition for rural oral health

Participants reported ongoing marginalization of oral health in rural community health planning, pointing to major gaps in advocacy and policy integration. They emphasized the absence of structured standards or initiatives for oral health promotion and highlighted its low visibility compared to conditions such as HIV/AIDS, which benefit from sustained prioritization and funding. While HIV-focused campaigns are vital, their dominance illustrates how competing health priorities overshadow oral health, despite its acknowledged role in overall well-being. This neglect reflects broader policy gaps at both national and provincial levels, where oral health has not been effectively integrated into non-communicable disease (NCDs) frameworks or community health promotion strategies. Participants emphasized this point, stating:

*“Currently*,* there are no established guidelines for tackling dental issues affecting the community*,* as oral health has never been prioritized. The only available initiative is centered on HIV awareness rather than oral health concerns” (C1P7).*

*“The sole campaigns currently in place are centered around HIV awareness*,* and there has never been a direction or standards for oral health within our community” (C3P5).*

#### Sub-theme 2.2: lack of community-based oral health programs

The findings from the FGDs highlight the lack of structured oral health programs in rural communities, revealing systemic neglect that contributes to the ongoing burden of oral disease. Participants consistently emphasized that while various health initiatives exist, especially those addressing HIV and home-based care, oral health has never been prioritized in community education or prevention efforts. This absence reflects a gap in health promotion strategies and highlights how oral health remains marginalized within the broader primary healthcare agenda. As one participant observed,

*“Even though there is a clinic [public primary health care clinic] in our community*,* we do not have any form of dental care benefits*,* as there are no initiatives focused on promoting or improving oral health. The clinic [public primary health care clinic] seems to prioritize other health issues*,* leaving oral health issues not attended” (C1P6).*

Another commented:

*“In my community*,* there has never been any form of a program focusing on oral health. Available programs are on home-based care and HIV awareness*,* but there is a lack of initiatives promoting oral health” (C5P3).*

#### Sub-theme 2.3: narrow scope of community health campaign

The findings of this study reveal a significant inequity in prioritization of health initiatives within rural communities. Participants consistently noted that campaigns addressing conditions such as HIV and elder care were well supported, adequately funded, and actively involved community healthcare workers (CHWs), locally referred to as ‘home-based care workers’. In contrast, oral health has been excluded from these initiatives, despite the potential to utilize existing structures for more comprehensive service delivery. This omission reflects ongoing fragmentation in health promotion, where oral health is separated from broader health agendas and regarded as peripheral to overall well-being. Such marginalization reflects the inefficient use of human resources and perpetuates the misconception that oral health is a secondary concern rather than an integral component of general health. The participants emphasized this point, noting:

*“Look*,* we already have home-based care workers looking after our grannies [elderly people] on a daily basis*,* and this initiative originated from the clinic [public PHC clinic]*,* so my question is*,* why can’t these people be used to do something for dental?. There is funding for this campaign. Oral health has never been supposed or included” (C1P4).*

*“We do have campaigns in our communities; older people usually benefit*,* but oral health is not part of it. Home-based care workers are employed to run this campaign*,* but it does not look like they are being used properly. Maybe oral health needs its own people*,* I do not know…” (C4P6).*

### Theme 3: intrinsic determinants of oral health

#### Sub-theme 3.1: knowledge of oral health self-care

##### Category 3.1.1: awareness of the link between oral and general health

Although some participants recognized a basic link between oral and general health, the findings revealed a limited understanding of the broader systemic implications of poor oral health for overall well-being. This partial awareness reinforced the perception of oral health as an isolated concern, disconnected from larger health priorities. Such views reflect the marginalization of oral health within broader health discourses, where weak integration into PHC and limited public health communication contribute to a fragmented understanding of oral–systemic connections. The lack of structured education and advocacy perpetuates community misconceptions, discourages preventive care-seeking, and sustains reliance on curative, pain-driven models of oral healthcare.

*“Painful tooth affects other parts of the body like having a headache. I am clueless about the connection between my mouth and the entire body. Maybe it’s different*,* I mean there is nothing much done about oral health*,* so it must be handled differently” (C2P4).*

*“For all I know is that the mouth is part of the body*,* so when there is a toothache*,* one experience a headache. I have no idea what happens when one has bad oral health. I do not know much about that; I am not good with this stuff” (C5P6).*

##### Category 3.1.2: awareness of oral health conditions

Dental caries and gingivitis were the most recognized oral conditions, with all participants reporting prior experience with at least one. This narrow awareness reflects a limited understanding of oral health, where recognition is primarily shaped by pain, bleeding, or visible tooth damage. Several participants also identified tonsillitis as an oral disease, suggesting blurred boundaries in the community’s understanding of oral–systemic conditions and highlighting gaps in oral health literacy. These accounts illustrate how community knowledge is based on personal and observable symptoms rather than biomedical definitions, revealing a disconnect between lay perceptions and professional discourses.

*“I am sure that you dental people have correct terms for oral conditions*,* but what I know is swollen gums and obviously rotten teeth” (C1P4).*

*“I do not know much…some of the oral diseases that I know of include tooth decay*,* tonsillitis*,* and bleeding” (C4P2).*

##### Category 3.1.3: daily routine of oral care

Interestingly, while the majority of participants demonstrated awareness of daily oral hygiene practices, particularly the utilization of toothbrushes and toothpaste, the depth of their knowledge was notably limited. Participants exhibited unfamiliarity with critical components of effective self-care, such as proper brushing techniques, the significance of fluoride in toothpaste, and the recommended frequency for toothbrush replacement. The continued adherence to traditional practices, including the use of miswak and charcoal, further exemplifies the coexistence of biomedical and cultural approaches to oral hygiene within this population. These findings indicate a partial yet fragmented understanding of oral health self-care, wherein routine practices are evident but are not necessarily guided by evidence-based recommendations. As one participant articulated:

*“I brush my teeth with a toothbrush and toothpaste twice daily*,* and I also use miswak as well as charcoal to make my teeth whiter. Well*,* I don’t know anything about a brushing technique…wait*,* what is a fluoride toothpaste?” (C4P2).*

*“I brush my teeth 2 times a day. I do not change my toothbrush. Well*,* that happens after it is lost or I used it for other purposes…but I do not see the need to change it. Please advise” (C2P9).*

##### Category 3.1.4: routine dental check-ups for oral health maintenance

Participants consistently reported that they used oral health services only during instances of acute pain, with little recognition of the value of preventive care. This pain-driven pattern reflects a focus on symptoms, where dental visits are primarily associated with extractions or temporary relief rather than long-term maintenance. Such behaviors highlight significant gaps in oral health literacy and the lack of community-based preventive education, both of which reinforce extraction-oriented models of care. The lack of consistent messaging from health professionals about the importance of routine visits further entrenches community perceptions of oral healthcare as crisis-driven rather than integral to holistic well-being. The participants reinforced this perception, explaining:

*“The only reason for my visit to the hospital was because I had a painful tooth; other than that*,* there is no reason why I should go there (C3P5).*

*“The reason for the visit was only for tooth removal*,* and that is the only time I ever visited the hospital with dental related issues. This may be because we have never been advised that regular visits are necessary (C2P1).*

#### Sub-theme 3.2: attitudes on oral health self-care

##### Category 3.2.1: prevention of oral health diseases

When participants reflected on the importance of oral health, they consistently identified daily self-care as the primary strategy for preventing oral disease. Their reflections demonstrated a strong awareness that neglecting oral hygiene not only accelerates disease progression but also leads to financial consequences when treatment becomes necessary. This emphasis shows a nuanced understanding of prevention as both a health-preserving and cost-saving measure, with regular tooth brushing as a central practice for protecting oral health. While participants acknowledged the structural barriers limiting access to professional care, their accounts highlighted an intrinsic recognition that consistent self-care provides a tangible and accessible means of alleviating the burdens of disease and treatment costs. As one participant commented:

*“I think the focus should be on taking good care of our teeth on a regular basis*,* as it is important to prevent cavities. If we do not*,* we might need to have the teeth pulled out…*,* and it is costly” (C3P8).*

Another added:

*“I think brushing with toothpaste on a daily basis is important to take care of our teeth and gums to prevent bad breath and tooth problems that can lead to tooth loss” (C4P3).*

##### Category 3.2.2: physical functionality and well-being

The findings emphasized participants’ strong commitment to preserving their natural teeth, which they viewed as essential for physical functionality. Teeth were described not only in aesthetic terms but also as crucial for daily activities like chewing, biting, and clear speech, all of which are vital to overall well-being. This perspective shows that community members see oral health as more than just the absence of disease; they consider it a prerequisite for maintaining their functional capacity and quality of life. As one participant explained,

*“I think it is very important to ensure that we keep our teeth against being pulled out*,* to allow us to bite*,* chew*,* and speak (C4P9).*

##### Category 3.2.3: social and psychological implications

Study participants recognized the psychosocial aspects of oral health, linking it not only to physical well-being but also to confidence, self-perception, and social participation. Poor oral health was seen as a source of embarrassment, strained relationships, and diminished self-esteem, emphasizing its impact on quality of life and interpersonal functioning. These views confirm that oral health goes beyond the absence of disease and includes identity, dignity, and social interaction. Such insights highlight the need for oral health promotion in rural communities to focus not only on clinical outcomes but also on the psychosocial benefits that contribute to well-being. As one participant reflected,

*“I have always believed that brushing twice daily brings an extra boost in confidence and has a way to improve interaction with other people (C1P5).*

#### Sub-theme 3.3: practices of oral health self-care

##### Category 3.3.1: promoting good oral hygiene habits

When reflecting on the prevention and management of oral diseases, participants revealed a paradox: despite limited oral health education and restricted access to professional care, they took initiative by making lifestyle changes. These practices centered on reducing sugary food intake and maintaining regular oral hygiene, especially brushing twice daily. They reflect resilience, as participants drew on limited knowledge and perceived risk to shape preventive behaviors without structured guidance. However, their uncertainty about the effectiveness of these efforts highlights the shortcomings of fragmented health communication strategies, leaving individuals without clear direction for effective prevention. The following excerpts illustrate this:

*“One lifestyle behavior that I have acquired is to ensure that I brush twice daily*,* although there is not much I know. Dental care is very scarce and expensive….” (C1P4).*

*“I do not really know how much that works*,* but lifestyle habits such as poor brushing are bad for our teeth as well as foods containing sugar. So*,* I decided to cut down such foods and maintain good brushing*,* which I am not entirely sure if it is correct (C5P3).*

##### Category 3.3.2: need for immediate oral healthcare

Despite participants’ commendable efforts to maintain oral health with limited knowledge, the findings reveal how systemic barriers compel communities to adopt harmful or suboptimal coping strategies. Restricted access to formal oral healthcare led some participants to undertake extreme measures, including self-tooth extraction and reliance on traditional healers for indigenous remedies. These practices illustrate the interplay between structural inequities in service delivery and community-level responses to unmet oral health needs. The absence of timely and accessible services not only sustains dependence on potentially unsafe alternatives but also exposes structural failures within the health system to deliver equitable and preventive care. Participants expressed this as follows:

*“It takes a long time to access dental services in our community*,* so I end up going to traditional healers for herbs to treat toothache (C5P3).*

*“I pulled most of my teeth out because I could bear pain due to a hassle when trying to find dental care (C1P5).*

## Discussion

The aim of this study was to explore community members’ perceptions of oral health in rural communities. A qualitative approach was adopted, using multiple FGDs to examine participants’ perceptions, understanding, and experiences of the quality and availability of oral health services. As outlined in the results, the five FGDs identified three prominent themes: barriers to oral healthcare access, inadequate oral health advocacy, and intrinsic determinants of oral health. Overall, these findings suggest that oral health remains neglected and is not prioritized to the same extent as other NCDs under universal health coverage in remote and rural communities [40].

### Barriers to oral healthcare access (Community level)

This study highlights that barriers to accessing oral healthcare in rural South African communities are deeply embedded in systemic challenges, illustrating the connections between community-level experiences and broader structural health system issues. Participants identified critical obstacles, including a shortage of oral health professionals, the absence or deterioration of mobile oral health services, financial and infrastructural barriers, ineffective referral systems, and limited oral health knowledge among nurses. Collectively, these challenges demonstrate how structural inequities perpetuate oral health disparities in underserved rural communities.

The shortage of OHPs identified in this study reflects global trends, where unequal distribution, inadequate retention incentives, and professional migration undermine access to preventive care and increase dependence on extractions [23, 61–63]. Participants’ concerns about the absence of mobile oral health services align with global evidence showing that such services improve coverage and access in underserved rural populations [64, 65], while their deterioration in this context reflects weak prioritization and infrastructure limitations [31].

Financial hardship and poor infrastructure further restricted access to oral healthcare, consistent with findings from SA and other low-resource settings [10, 12, 31, 66, 67]. The inefficiency of referral systems observed in this study echoes earlier local evidence, which similarly noted that poor referral mechanisms hinder the integration of oral health into PHC [68]. Participants’ reports of limited oral health knowledge among nurses are consistent with the literature, indicating that inadequate training constrains timely prevention, early detection, and comprehensive patient education [68, 69].

The findings of this study emphasize the need for targeted interventions at both the policy and practice levels. First, workforce shortages require specific incentives to recruit and retain OHPs in rural communities [61]. Second, mobile oral health services should be reintroduced, maintained, and integrated into community engagement programs to overcome geographical and financial barriers [70]. Third, oral health must be fully incorporated into PHC through interprofessional collaboration with strengthened referral pathways to improve continuity of care and promote efficient resource utilization [71]. Fourth, integrating oral health education into nursing curricula is critical for equipping frontline providers to support the prevention and early detection of oral diseases [72]. Finally, broader system-level responses, such as financial support for low-income families and infrastructure development, are necessary to address the social determinants of oral health and advance equity in service delivery [73]. The findings of this study provide valuable insights into persistent structural barriers and inform recommendations that can guide policies and practices to promote oral health equity in rural contexts.

### Inadequate oral health advocacy (National/Systemic level)

The results of this study highlight the historical neglect of oral health within SA’s national health priorities, significantly impacting rural communities. This oversight has led to fragmented service delivery, hindered progress toward universal health coverage, and relegated rural populations to passive recipients of inadequate or non-existent oral health services. Participants revealed a substantial gap in oral health advocacy in rural SA, reflected in the inadequate incorporation of oral health into broader NCD frameworks, where initiatives such as HIV awareness receive greater priority. This lack of prioritization has constrained the visibility and resources allocated to oral health, particularly in underserved rural settings. The findings also highlight the need for accessible community-based oral health programs and the potential role of CHWs in strengthening rural service delivery.

These results align with the existing literature, which highlights the marginalization of oral health in health policy agendas. Foláyan and colleagues have similarly called for systematic policy revisions to embed oral health within broader health promotion and disease prevention strategies [40, 74]. Global guidance from the WHO reinforces this perspective, recommending the establishment of robust national coordinating entities, workforce planning to address shortages and maldistribution, and the integration of digital innovations, such as teledentistry, to improve access [75]. The findings of this study also resonate with evidence from other low-resource settings, where community-based oral health programs have demonstrated economic and accessibility benefits [27, 75]. Moreover, consistent with prior reports [76–80], this study reveals both the potential and limitations of CHWs. While they can expand basic oral health coverage cost-effectively, their limited training and inconsistent government support raise concerns about sustainability, quality, and effectiveness.

The findings of this study demonstrate the pressing necessity for policy reforms that integrate oral health into SA’s comprehensive health strategies. It is imperative to revise the National Oral Health Policy to integrate oral health within frameworks addressing NCDs and PHC delivery, thereby ensuring equity in rural contexts [81, 82]. Enhanced community-based oral health programs have the potential to alleviate travel burdens, improve preventive measures, and reduce the indirect costs associated with seeking care [27, 76, 77]. Furthermore, CHWs may serve as a viable workforce solution, provided they receive structured training, ongoing professional development, and sustained governmental support [78, 83, 84] . Although this study did not investigate national resource allocation patterns or the processes of policy implementation, its insights reveal how insufficient advocacy, inadequate policy integration, and the underutilization of community-level strategies collectively perpetuate disparities in access to oral healthcare. Addressing these systemic and community-level deficiencies is crucial for advancing universal health coverage and fulfilling SA’s commitment to the SDGs.

### Intrinsic determinants of oral health (Individual level)

This study elucidates the intricate interplay between knowledge, attitudes, and practices related to oral health self-care within rural South African communities, revealing both strengths and significant deficiencies in community oral health self-care practices. Participants exhibited limited oral health literacy, with their comprehension of the connection between oral and general health primarily restricted to associating tooth pain with headaches, and their awareness of dental conditions chiefly limited to visible issues such as tooth decay and gingival swelling. Although daily tooth brushing and dietary moderation were frequently reported, these practices were often compromised by reliance on traditional products, inadequate awareness of fluoride, and irregular replacement of toothbrushes, indicating a lack of certainty and insufficient professional guidance.

Concurrently, participants conveyed positive attitudes toward self-care, acknowledging its preventive potential in reducing cavities, extractions, and associated costs, while also recognizing the functional and psychosocial benefits of maintaining oral health. However, access to professional oral health services remained predominantly reactive and problem-driven, with extractions pursued primarily for pain relief, and in some instances, reliance on traditional remedies or self-extraction.

These findings align with evidence from SA and other low-resource contexts, wherein limited awareness of fluoridated products and preventive behaviors contributes to a heightened burden of oral disease [85–87]. The reliance on pain-driven service utilization reflects structural inequities and underdeveloped community-based oral health initiatives, while the persistence of traditional remedies and self-extraction mirrors patterns observed in various other African settings [35, 88, 89]. Simultaneously, participants’ acknowledgment of the preventive, functional, and psychosocial value of oral health resonates with the WHO’s holistic definition, as well as with international literature that links oral health to quality of life and social participation [1]. Together, these findings illustrate the paradox of rural communities valuing oral health while systemic inequities and limited guidance prevent the translation of positive attitudes into sustainable practices.

The findings highlight the need to enhance community-based oral health education, ideally integrated within PHC frameworks, to facilitate the conversion of positive attitudes into effective behaviors [27, 90]. Expanding school-based oral health programs, ensuring affordable access to fluoride toothpaste, and training CHWs to deliver contextually relevant health promotion can mitigate harmful practices and enhance the uptake of preventive care [90, 91]. Addressing workforce shortages and improving rural service infrastructure remain critical to diminishing inequities and aligning oral health initiatives with universal health coverage objectives [40]. Nonetheless, the findings offer valuable insights into the interplay between knowledge, attitudes, and practices in rural communities, illustrating how systemic barriers and limited literacy impede oral health outcomes. These insights serve as a foundation for targeted interventions aimed at bolstering oral health equity in rural SA.

### Limitations

The current study has several limitations that warrant acknowledgment. It employed a convenience sample drawn from selected rural communities in the Vhembe District, with the findings reflecting the perspectives of participants within these specific contexts. As is characteristic of qualitative research, the results are not intended to be statistically generalizable, yet they may provide transferable insights for analogous rural and low-resource settings. While data saturation was achieved, the FGDs may not reflect the full diversity of perspectives. Reliance on self-reported accounts also introduces the possibility of social desirability bias. Nevertheless, the lived experiences of the participants convey systemic barriers that are likely to resonate with other rural communities facing similar oral health challenges.

### Recommendations

This study highlights the necessity for coordinated interventions at clinical, public health, and health system levels to mitigate oral health inequities in rural SA. Clinically, enhancing the oral health capacity of PHC providers and integrating preventive, patient-centered approaches, supported by digital innovations such as teledentistry, could improve early detection and decrease reliance on extractions. At the public health level, culturally tailored community programs, the mobilization of CHWs, and sustainable mobile oral health services are essential for enhancing awareness, prevention, and access in underserved areas.

At the systems level, revising the National Oral Health Policy to integrate oral health into NCD frameworks, strengthen workforce retention, and invest in rural infrastructure is essential for advancing equity, achieving universal health coverage, and aligning with the SDGs. Future research should focus on implementation studies, policy analyses, and community-based participatory approaches to evaluate the feasibility, effectiveness, and sustainability of these strategies, thereby generating context-specific evidence to guide long-term reforms.

## Conclusion

The findings of this study illuminate key factors contributing to disparities in access to oral health services in rural communities. Both intrinsic and extrinsic determinants exacerbate the burden of oral diseases and negatively affect the quality of life of rural residents. Addressing these challenges requires healthcare system service delivery models that are responsive to the specific needs of rural populations [35]. The study provides insights into the factors that OHPs and other relevant stakeholders should consider when developing community-based oral health programs. It also draws attention to the distribution of OHPs in rural communities, aligning with the objectives of SA’s National Oral Health Policy, which emphasizes preventive and oral health promotion.

## Supplementary Information

Supplementary Material 1: S1 Text. Consolidated Criteria for Reporting Qualitative Research (COREQ) checklist. S2 Text. Focus group discussion interview guide.

## Data Availability

The authors confirm that the data supporting the findings of this study is available upon request from the corresponding author.

## Abbreviations

AIDS: Acquired Immune Deficiency Syndrome
CHC: Community Health Center
CHW: Community Healthcare Worker
COREQ: Consolidated Criteria for Reporting Qualitative Research
FGD: Focus Group Discussion
HIV: Human Immunodeficiency Virus
NCD: Non-Communicable Disease
OHP: Oral Health Professional
PHC: Primary Health Care
SA: South Africa
SDG: Sustainable Development Goal
WHO: World Health Organization
