# Supplementary material for: Exploring community members’ perceptions of oral health in rural South Africa
Source: BMC Oral Health. 2025 Dec 7;26:72. doi: 10.1186/s12903-025-07490-1 (PMC12797676; doi:10.1186/s12903-025-07490-1)
Supplement: Supplementary file 1 — Supplementary Material 1: S1 Text. Consolidated Criteria for Reporting Qualitative Research (COREQ) checklist. S2 Text. Focus group discussion interview guide. [file 12903_2025_7490_MOESM1_ESM.pdf]

EXPLORING COMMUNITY MEMBERS' PERCEPTIONS OF ORAL HEALTH IN RURAL SOUTH AFRICA

Consolidated Criteria for Reporting Qualitative Studies (COREQ): 32-Item Checklist

**DOMAIN 1: RESEARCH TEAM AND REFLEXIVITY**		
**No. Item**	**Guide questions/description**	**Reported on Page #**
**Personal Characteristics**		
1. Inter viewer/facilitator	Which author/s conducted the interview or focus group?	Focus groups were conducted by primary author (HAN), assisted by 5 community caregiver leaders (research assistants)<br><br>Page 9
2. Credentials	What were the researcher's credentials? E.g. PhD, MD	HAN is a dental educator, whereas KJR and RM are senior dental educators. HAN is an oral hygienist with an MMedSc, while KJR and RM are dentists with MCHD and PhD, respectively. HAN is a PhD candidate, while Both KJR and RM are Associate Professors.<br><br>Page 7
3. Occupation	What was their occupation at the time of the study?	At the time of the study, HAN was affiliated with both the University of the Western Cape as a PhD student and Tshwane University of Technology as an academic. Both KJR and RM were affiliated with the University of the Western Cape, with KJR as a community dentistry specialist and RM as a deputy dean of Academic (Teaching and learning).<br><br>Page 1
4. Gender	Was the researcher male or female?	Research team contains 1 male (HAN) and 2 female (KJR and RM)<br><br>Page 7
5. Experience and training	What experience or training did the researcher have?	The researchers have significant experience conducting qualitative research and dental education and public health studies.<br><br>Page 7
**Relationship with participants**		

6. Relationship established	Was a relationship established prior to study commencement?	No prior relationships between researchers and participants.<br><br>Page 7
7. Participant knowledge of the interviewer	What did the participants know about the researcher? e.g. personal goals, reasons for doing the research	The participants were informed that the researcher was conducting a study on perceptions of oral health. The study's objectives, which included improving rural oral health, improving workforce distribution, and establishing a foundation for co-designing community oral health programs, were communicated to the participants as part of the pre-consent information.<br><br>Page 9
8. Interviewer characteristics	What characteristics were reported about the inter viewer/facilitator? e.g. Bias, assumptions, reasons and interests in the research topic	Although experienced in qualitative research methodologies, HAN may have subjected the study to bias due to his prior residence and strong interest in the oral health. However, KJR and RM provided expertise to enhance the credibility and counterbalance the potential insider bias.<br><br>Page 7
**DOMAIN 2: STUDY DESIGN**		
**Theoretical framework**		
9. Methodological orientation and Theory	What methodological orientation was stated to underpin the study? e.g. grounded theory, discourse analysis, ethnography, phenomenology, content analysis	The study employed a combined approach incorporating the general inductive approach described by Thomas (2006) and a three-stage process for qualitative data analysis by Williams & Moser (2019).<br><br>Page 10
**Participant selection**		
10. Sampling	How were participants selected? e.g. purposive, convenience, consecutive, snowball	A homogeneous convenience sampling approach was used to recruit adult members from five rural communities, with participation facilitated by collaboration with community caregiver leaders. Although caregiver leaders facilitated recruitment, the study was best characterised as convenience sampling, as participation relied entirely on voluntary responses to open invitations.<br><br>Page 8
11. Method of approach	How were participants approached? e.g. face-to-face, telephone, mail, email	Participants were recruited voluntarily through word of mouth, telephone invitations, posters, and flyers distributed across communities.

		Page 8
12. Sample size	How many participants were in the study?	50 participants (33 women, 17 men)<br><br>Page 8
13. Non-participation	How many people refused to participate or dropped out? Reasons?	None.
**Setting**		
14. Setting of data collection	Where was the data collected? e.g. home, clinic, workplace	Data were collected in five geographically distinct rural communities on the outskirts of Malamulele—Gijana (Magona), Govhu, Lombard, Mashobye, and Nghomunghomu<br><br>Page 6
15. Presence of non-participants	Was anyone else present besides the participants and researchers?	Yes, five community caregiver leaders were used as research assistants, one from each community.<br><br>Page 7
16. Description of sample	What are the important characteristics of the sample? e.g. demographic data, date	50 individuals participated (33 women and 17 men; aged 21–65 years, including 15 who had not completed secondary school). The sample comprised unemployed youth, working-age adults, and community elders, providing a broad perspective on oral health practices and the challenges they<br><br>8-9
**Data collection**		
17. Interview guide	Were questions, prompts, guides provided by the authors? Was it pilot tested?	A semi-structured discussion guide, informed by a driver diagram, was employed to explore oral health perceptions in rural communities, ensuring that discussions remained focused on key determinants and experiences. The guide was reviewed by the research team prior to use to confirm its clarity, relevance, and acceptability for the study context.<br><br>Page 9
18. Repeat interviews	Were repeat interviews carried out? If yes, how many?	No.
19. Audio/visual recording	Did the research use audio or visual recording to collect the data?	Discussions were audio-recorded with permission and transcribed verbatim.

		Page 9
20. Field notes	Were field notes made during and/or after the inter view or focus group?	Field notes were taken by community caregiver leaders (research assistants) during the focus groups.<br><br>Page 7
21. Duration	What was the duration of the interviews or focus group?	Focus groups lasted approximately 45 minutes<br><br>Page 9
22. Data saturation	Was data saturation discussed?	The data saturation was not discussed. However, data saturation was defined as the point at which no new concepts or perspectives emerged, as the study sought breadth of views rather than theoretical saturation. Analytical rigor was ensured through systematic coding, collaborative theme development and integration with existing literature.<br><br>Page 9
23. Transcripts returned	Were transcripts returned to participants for comment and/or correction?	Due to time constraints, transcripts were returned to trained community caregivers who documented group dynamics and nonverbal cues, and the transcripts were also verified by selected participants (member checking). However, structured coding and cross-researcher verification were used to ensure accuracy.<br><br>Page 11
**DOMAIN 3: ANALYSIS AND FINDINGS**		
**Data analysis**		
24. Number of data coders	How many data coders coded the data?	One external independent coder and multiple researchers (HAN, KJR, RM) collaboratively coded the data.<br><br>Page 11
25. Description of the coding tree	Did authors provide a description of the coding tree?	A coding framework was developed based on prior research and refined through iterative coding.<br><br>Page 10
26. Derivation of themes	Were themes identified in advance or derived from the data?	Themes were derived from the data through thematic analysis.<br><br>Page 10
27. Software	What software, if applicable, was used to manage the data?	ATLAS.ti software<br><br>Page 10

28. Participant checking	Did participants provide feedback on the findings?	The selected participants endorsed the findings<br><br>Page 11
**Reporting**		
29. Quotations presented	Were participant quotations presented to illustrate the themes/findings? Was each quotation identified? e.g. participant number	Participant quotations are presented in the results section and labelled by community number and participant number.<br><br>Page 11-26
30. Data and findings consistent	Was there consistency between the data presented and the findings?	Findings are supported by direct quotes.<br><br>Page 11-26
31. Clarity of major themes	Were major themes clearly presented in the findings?	Three major themes (1) barriers to oral healthcare access, (2) inadequate oral health advocacy, and (3) intrinsic determinants of oral health are clearly outlined in the result section.<br><br>Page 11-26
32. Clarity of minor themes	Is there a description of diverse cases or discussion of minor themes?	Sub-themes and categories are clearly outlined in the result sections and discussed within the major themes.<br><br>Page 11-26

FOCUS GROUP DISCUSSION PROTOCOL

Title of Research Project: A framework for oral health community engagement: A case study of the Vhembe district in Limpopo Province, South Africa

Focus Group Discussion Guide

Q1. In your view, why do you think it is important to take good care of your teeth or oral health?

a) What motivates you or others in your community to maintain oral health?
b) What challenges make it difficult to prioritise oral health?

Q2. What methods do you or your community members use to clean your mouth?

a) What materials or tools do you use?
b) How often are these practices followed?

Q3. How many times have you visited this clinic, hospital, or health centre for oral health care?

a) What was the main reason for your most recent visit?
b) Are you aware of oral health services delivered through mobile clinics?
c) How easy or difficult is it for you to access these services?
d) Are there enough facilities providing oral health care in your community?
e) How would you describe the condition of facilities where oral health services are provided?
f) What are the most significant challenges you face when trying to access oral health services?
g) Are there any policies or initiatives you think could address these barriers?
h) How does the cost of oral health services affect people's decisions to seek care?

Q4. What oral diseases do you know of?

a) What do you think causes these conditions?

Q5. How do you or others in your community prevent or treat oral diseases?

Q6. Do you think oral health and general health are connected?

a) If yes, how can oral health affect the overall health of the body?

Q7. What kinds of lifestyle practices do you think are harmful to oral health?

Thank you for your participation
